# Association between skull bone mineral density and periodontitis: Using the National Health and Nutrition Examination Survey (2011–2014)

**DOI:** 10.1371/journal.pone.0271475

**Published:** 2022-12-30

**Authors:** Fuqian Jin, Jukun Song, Yi Luo, Beichuan Wang, Ming Ding, Jiaxin Hu, Zhu Chen

**Affiliations:** 1 School of Stomatology, Zunyi Medical University, Zunyi, Guizhou, China; 2 Department of Oral Medicine, Guiyang Hospital of Stomatology, Guiyang, Guizhou, China; 3 The Affiliated Stomatological Hospital & Stomatology of Guizhou Medical University, Guizhou Medical University, Guiyang, China; University of Life Sciences in Lublin, POLAND

## Abstract

**Background and objective:**

Bone mineral density (BMD) and periodontitis have been the subject of many studies. However, the relationship between skull (including mandible) BMD and periodontitis has not been extensively studied. An objective of this cross-sectional study was to examine the relationship between skull BMD and periodontitis using data from the National Health and Nutrition Examination Surveys (NHANES) for 2011–2012 and 2013–2014.

**Materials and methods:**

From NHANES 2011–2014, 3802 participants aged 30–59 were selected. We divided the skull BMD level into quartiles to check the distribution of variables. Periodontitis was defined by the Centers for Disease Control and Prevention (CDC) and the American Association of Periodontology (AAP) in 2012. Multivariate logical regression analysis was used to explore the independent relationship between skull BMD and periodontitis. The generalized additive model (GAM), smooth curve fitting (penalty spline) and threshold effect analysis was used to evaluate dose-response relationship between skull BMD and periodontitis and the potential nonlinear relationship between skull BMD and periodontitis. Finally, subgroup analysis and interaction test were conducted to determine the role of covariates between skull BMD and periodontitis.

**Results:**

The overall average skull BMD of 3802 participants was 2.24g/cm2, the average age was 43.94 years, and the prevalence of periodontitis was 41.03%. In the fully adjusted logistic regression model, skull BMD and periodontitis showed an independent negative correlation (OR 0.73, 95% CI 0.59–0.90, P = 0.0032) and a linear relationship. Compared with the lowest quartile array (Q1:1.22–1.98) of skull BMD, the highest quartile array(Q4: 2.47–3.79) had a significantly lower risk of periodontitis (OR 0.70,95% CI 0.56–0.87, P = 0.0014). Subgroup analysis showed a highly consistent negative correlation between skull BMD and periodontitis. In the interaction test, people with moderate poverty income ratio (1.57–3.62) and those who had more than 12 alcohol drinks in the past year had a lower risk of periodontitis.

**Conclusions:**

This result suggested that periodontal disease can be related to low skull BMD, for those people, oral hygiene and health care should be more closely monitored. Validation of our findings will require further research.

## Introduction

The burden of periodontitis continues to be a worldwide public health problem, and the majority of periodontitis incidence is observed in those between 55 and 59 years of age, while younger people are experiencing an increasing incidence of periodontitis [1]. Periodontitis is chronic inflammation of the tissue supporting teeth, and if it progresses, it can lead to alveolar bone loss and ultimately tooth loss [2]. Periodontitis occurs when dental plaque accumulates on the teeth and results in an imbalance between bacterial invasion and host defense [3]. Meanwhile, host responses to general health conditions as well as be associated with periodontitis [4, 5]. For example, osteoporosis and periodontitis have been associated in most cross-sectional studies, especially for postmenopausal women [6–10].

Bone mineral density (BMD) measures are the optimal method for diagnosing osteoporosis and osteopenia. The World Health Organization recommends using dual energy X-ray absorptiometry (DXA) to assess the BMD of the spine, hip, and forearm [11]. Apparently, osteoporosis has been associated with the risk of periodontal diseases [12]. Human osteoporosis may negatively impact alveolar bone height, but periodontitis in local oral cavity will not cause bone health in the whole body [13]. A study by Munhoz et al. documented the BMD of mandible was measured by DXA in systemically healthy subjects and then found that the low BMD of mandible may be related to chronic periodontitis [14]. The mandible has high bone turnover, increased blood flow, and is more sensitive to osteoclast and osteoblast activity than any other sites, however, researchers encountered difficulties working with the mandible because of its complex bone mineral distribution [11]. For example, the BMD of the anterior teeth and premolar area of the mandible is higher than that of the molar area, and even the BMD of any part of the mandible is higher than that of all parts of the maxilla [15]. Meanwhile, the skull is connected by irregular shapes and thickness of the bones, fibrous joints, and complex muscle relationships, which seems to rule out the possibility of analyzing a single bone [16]. The skull (including mandible) BMD, which as part of the whole-body BMD measurement, is well correlated to the rest of the skeleton [17]. Due to very little mechanical strain and weight bearing on the skull, it is a unique part of the skeleton, at the same time, the measurement of skull BMD may be used to screen for hereditary diseases, skeletal artifacts, or to assess oral bone loss [17, 18]. Athlestes with stress sites in their skeleton can increase bone density through impact loading sports. The study by Courteix et al. [19] reported that the skull BMD in gymnasts is lower than other people because of the absence of stress. It is possible to screen for osteoporosis by measuring BMD with axial skull CT because patients with a positive head CT scan for the condition are twice as likely to suffer fractures as healthy people are [20].

Research on the relationship between skull BMD and periodontitis in a large and representative population is necessary to develop. Due to this, we analyzed secondary data based on available data from the National Health and Nutrition Examination Survey (NHANES). Study objectives are to determine if there is a significant relationship between skull BMD and periodontitis and to understand the associated confounders.

## Materials and methods

### Data source

In the present cross-sectional retrospective analysis, continuous NHANES data from cycles 2011–2012 and 2013–2014 were analyzed. The Centers for Disease Control and Prevention (CDC) manages the National Center for Health Statistics (NCHS) which conducts the NHANES to assess the health and nutritional status of children, adults, and the elderly. All the NHANES protocols were approved by the National Center for Health Statistics ethics review board, and written informed consent was obtained from all participants [21]. This modeling investigation was exempt from review because it used published deidentified data sets that included no personally identifiable information. The data from the CDC website can be downloaded for free (https://wwwn.cdc.gov/nchs/nhanes/Default.aspx). This manuscript meets the criteria stated at the STROBE guidelines.

## Study population

There were 19931 participants in NHNAES 2011–2012 and 2013–2014. NHANES examined the skull BMD of participants aged 8–59 years old, and examined the full mouth periodontium of participants older than 30 years old. Therefore, the final age of participants was 30–59 years old. First of all, we selected the participants who were completely evaluated skull BMD (n = 10104), of whom 4123 participants had a full mouth periodontal examination. Then, those patients with incomplete clinical or sociodemographic data were deleted. Finally, in this study, 3802 of 19931 participants were screened.

### Assessment of skull BMD and covariates

The independent variable in the present study was the skull BMD (g/cm^2^), which with Apex 3.2 software, scans were acquired on the Hologic Discovery model A densitometers (Hologic, Inc, Bedford, Massachusetts). The following participants are excluded from DXA examination: ① Pregnant women; ② Self report of using barium radiocontrast agent in the past 7 days; ③ Self-reported weight over 450 pounds or height over 6 feet 5 inches. Covariates include demographic, examination, and questionnaire variables. Demographic variables include sex, age, race, education level (high school and below; more than high school), and poverty income ratio (PIR, low (0–1.56), middle (1.57–3.62), high (3.63–5), triquantile). Examination variables include handgrip strength (kg) (low (10.1–31.7), middle (31.75–42.55), high (42.6–84.8), triquantile), body mass index (BMI, <24.9 kg/m^2^, 25.0–29.9 kg/m^2^, > = 30.0 kg/m^2^) [22]. The questionnaire variables include smoking consumption (smoked at least 100 cigarettes in life?); Alcohol consumption(had at least 12 alcohol drinks/1yr?); The doctor diagnosed hypertension, diabetes and hypertriglyceridemia; General health condition (excellent/very good/good, fair/poor); Depressive symptoms are defined as the frequency of nine depressive symptoms using PHQ-9 [23]; We use the best sleep time reported in the literature to divide the sleep time: <7h and > = 7h [24]; Frequency of interdental hygiene in the past 7 days (0; 1–4; 5–7).

### Assessment of periodontitis

The target dependent variable was periodontitis (dichotomous variable). The mobile examination center (MEC) was used for periodontal examination of participants aged 30 years and above. For oral health examinations, the NHANES operating manual describes the training and calibration process [25]. According to the 2012 CDC/American Association of Periodontology (AAP) case definition of periodontitis, participants must have at least two teeth that meet the specific detection threshold, and periodontitis is divided into four grades: no, mild, moderate and severe periodontitis [26]. Mild periodontitis was defined as ≥2 interproximal sites with AL ≥3mm and ≥2 interproximal sites with PD ≥4 mm (not on same tooth) or one site with PD ≥5 mm. Moderate periodontitis was defined as ≥2 interproximal sites with AL ≥4 mm (not on same tooth), or ≥2 interproximal sites with PD ≥5 mm (not on same tooth). Severe periodontitis was defined as ≥2 interproximal sites with AL ≥6 mm (not on same tooth) and ≥1 interproximal site with PD ≥5 mm. The final number of periodontitis cases is the sum of mild, moderate and severe cases.

### Statistical analysis

Data are expressed as mean ± standard deviation or ratio. Kruscal Whillis H test (continuous variable) and chi square test (categorical variable) are used to determine whether there is statistical difference between different skull BMD groups (quartile). We used unadjusted and multivariable adjusted logistic regression analysis to assess the independent association between skull BMD and periodontitis. Crude model does not adjust covariates; Model Ⅰ only adjusts sociodemographic data; Model Ⅱ adjusts all covariates. We convert skull BMD to quartile and calculate the trend of P for sensitivity analysis to ensure the robustness of data analysis. At the same time, the generalized additive model (GAM) and smooth curve fitting (penalty spline), and threshold effect analysis were used to evaluate dose-response relationship and the potential nonlinear relationship between skull BMD as a continuous variable and periodontitis. Finally, subgroup analysis and interaction test were conducted to determine the role of covariates between skull BMD and periodontitis. All the analyses were performed with the statistical software packages R (http://www.R-project.org, The R Foundation) and Empower Stats (http://www.empowerstats.com, X&Y Solutions, Inc, Boston, MA). All tests were two-sided and P values lower than 0.05 were considered statistically significant.

## Results

### Baseline characteristics of participants

The population characteristics and other covariates according to the quartile distribution of skull BMD are shown in Table 1. A total of 3802 participants were included in this study. In general, the average skull BMD was 2.24g/cm^2^, and the average age of participants was 43.94 years, and 41.03% suffered from periodontitis. No significant differences in education level, PIR, alcohol consumption, hypertriglyceridemia, general health condition, depression symptoms, hours of sleep, and frequency of interdental hygiene (last 7 days) were detected in the quartile of skull BMD. The subjects in the group with the highest skull BMD (Q4: 2.47–3.79g/cm^2^) are more likely to be younger, female, non-Hispanic black, with higher BMI, lower handgrip strength, less smoking (less than 100 cigarettes), no hypertension and diabetes, and healthier periodontium (P<0.05).

**Table 1:** Baseline characteristics of participants (N = 3802).

Skull BMD, g/cm^2^ quartile		Total	Q1(1.22–1.98)	Q2(1.98–2.21)	Q3(2.21–2.47)	Q4(2.47–3.79)	P-value
N		3802	951	945	955	951	
Age, (years, mean ± SD)		43.94 ± 8.54	44.81 ± 9.11	43.80 ± 8.63	43.37 ± 8.36	43.78 ± 7.98	0.002
Sex, n (%)							<0.001
	Male	1907 (50.16%)	628 (66.04%)	555 (58.73%)	431 (45.13%)	293 (30.81%)	
	Female	1895 (49.84%)	323 (33.96%)	390 (41.27%)	524 (54.87%)	658 (69.19%)	
Race/Ethnicity, n (%)							<0.001
	Mexican American	504 (13.26%)	149 (15.67%)	137 (14.50%)	103 (10.79%)	115 (12.09%)	
	Other Hispanic	347 (9.13%)	103 (10.83%)	85 (8.99%)	76 (7.96%)	83 (8.73%)	
	Non-Hispanic White	1477 (38.85%)	372 (39.12%)	408 (43.17%)	380 (39.79%)	317 (33.33%)	
	Non-Hispanic Black	798 (20.99%)	109 (11.46%)	125 (13.23%)	234 (24.50%)	330 (34.70%)	
	Other Race-Including Multi-Racial	676 (17.78%)	218 (22.92%)	190 (20.11%)	162 (16.96%)	106 (11.15%)	
Education Level, n (%)							0.129
	High school and below	1494 (39.29%)	396 (41.64%)	384 (40.63%)	355 (37.17%)	359 (37.75%)	
	More than high school	2808 (60.71%)	555 (58.36%)	561 (59.37%)	600 (62.83%)	592 (62.25%)	
PIR, n (%)							0.659
	Low (0–1.56)	1253 (32.96%)	328 (34.49%)	315 (33.33%)	298 (31.20%)	312 (32.81%)	
	Middle (1.57–3.62)	1273 (33.48%)	310 (32.60%)	302 (31.96%)	338 (35.39%)	323 (33.96%)	
	High (3.63–5)	1276 (33.56%)	313 (32.91%)	328 (34.71%)	319 (33.40%)	316 (33.23%)	
BMI, n (%)							<0.001
	< = 24.9	1049 (27.59%)	322 (33.86%)	257 (27.20%)	262 (27.43%)	208 (21.87%)	
	25.0–29.9	1325 (34.85%)	391 (41.11%)	370 (39.15%)	307 (32.15%)	257 (27.02%)	
	> = 30.0	1428 (37.56%)	238 (25.03%)	318 (33.65%)	386 (40.42%)	486 (51.10%)	
Handgrip strength, n (%)							<0.001
	Low (10.1–31.7)	1260 (33.14%)	274 (28.81%)	267 (28.25%)	343 (35.92%)	376 (39.54%)	
	Middle (31.75–42.55)	1269 (33.38%)	298 (31.34%)	314 (33.23%)	303 (31.73%)	354 (37.22%)	
	High (42.6–84.8)	1273 (33.48%)	379 (39.85%)	364 (38.52%)	309 (32.36%)	221 (23.24%)	
Smoking consumption, n (%)							0.003
	Yes	1592 (41.87%)	427 (44.90%)	413 (43.70%)	400 (41.88%)	352 (37.01%)	
	No	2210 (58.13%)	524 (55.10%)	532 (56.30%)	555 (58.12%)	599 (62.99%)	
Alcohol consumption, n (%)							0.062
	Yes	2725 (71.67%)	681 (71.61%)	691 (73.12%)	695 (72.77%)	658 (69.19%)	
	No	850 (22.36%)	200 (21.03%)	202 (21.38%)	204 (21.36%)	244 (25.66%)	
	Unknow	227 (5.97%)	70 (7.36%)	52 (5.50%)	56 (5.86%)	49 (5.15%)	
Hypertension, n (%)							0.002
	Yes	1060 (27.88%)	227 (23.87%)	253 (26.77%)	291 (30.47%)	289 (30.39%)	
	No	2742 (72.12%)	724 (76.13%)	692 (73.23%)	664 (69.53%)	662 (69.61%)	
Hypertriglyceridemia, n (%)							0.057
	Yes	1178 (30.98%)	325 (34.17%)	296 (31.32%)	275 (28.80%)	282 (29.65%)	
	No	2624 (69.02%)	626 (65.83%)	649 (68.68%)	680 (71.20%)	669 (70.35%)	
Diabetes, n (%)							0.022
	Yes	402 (10.57%)	91 (9.57%)	93 (9.84%)	92 (9.63%)	126 (13.25%)	
	No	3400 (89.43%)	860 (90.43%)	852 (90.16%)	863 (90.37%)	825 (86.75%)	
General health condition, n (%)							0.566
	Excellent/Very good/Good	3067 (80.67%)	777 (81.70%)	751 (79.47%)	765 (80.10%)	774 (81.39%)	
	Fair/Poor	735 (19.33%)	174 (18.30%)	194 (20.53%)	190 (19.90%)	177 (18.61%)	
Depressive symptoms, n (%)							0.641
	Yes	300 (7.89%)	66 (6.94%)	76 (8.04%)	79 (8.27%)	79 (8.31%)	
	No	3274 (86.11%)	818 (86.01%)	815 (86.24%)	819 (85.76%)	822 (86.44%)	
	Unknow	228 (6.00%)	67 (7.05%)	54 (5.71%)	57 (5.97%)	50 (5.26%)	
Hours of sleep, n (%)							0.16
	<7h	1622 (42.66%)	405 (42.59%)	400 (42.33%)	385 (40.31%)	432 (45.43%)	
	> = 7h	2180 (57.34%)	546 (57.41%)	545 (57.67%)	570 (59.69%)	519 (54.57%)	
Frequency of Interdental Hygiene (last 7 days), n (%)							0.096
	0	1127 (29.64%)	318 (33.44%)	279 (29.52%)	275 (28.80%)	255 (26.81%)	
	1–4	1282 (33.72%)	302 (31.76%)	323 (34.18%)	322 (33.72%)	335 (35.23%)	
	5–7	1393 (36.64%)	331 (34.81%)	343 (36.30%)	358 (37.49%)	361 (37.96%)	
Periodontitis, n (%)							<0.001
	Yes	1560 (41.03%)	446 (46.90%)	407 (43.07%)	364 (38.12%)	343 (36.07%)	
	No	2242 (58.97%)	505 (53.10%)	538 (56.93%)	591 (61.88%)	608 (63.93%)	

Abbreviations: PIR, poverty income ratio. BMI, body mass index.

^#^Periodontitis contained mild, moderate, and severe periodontitis.

## Relationship between skull BMD and periodontitis

**Fig 1:**
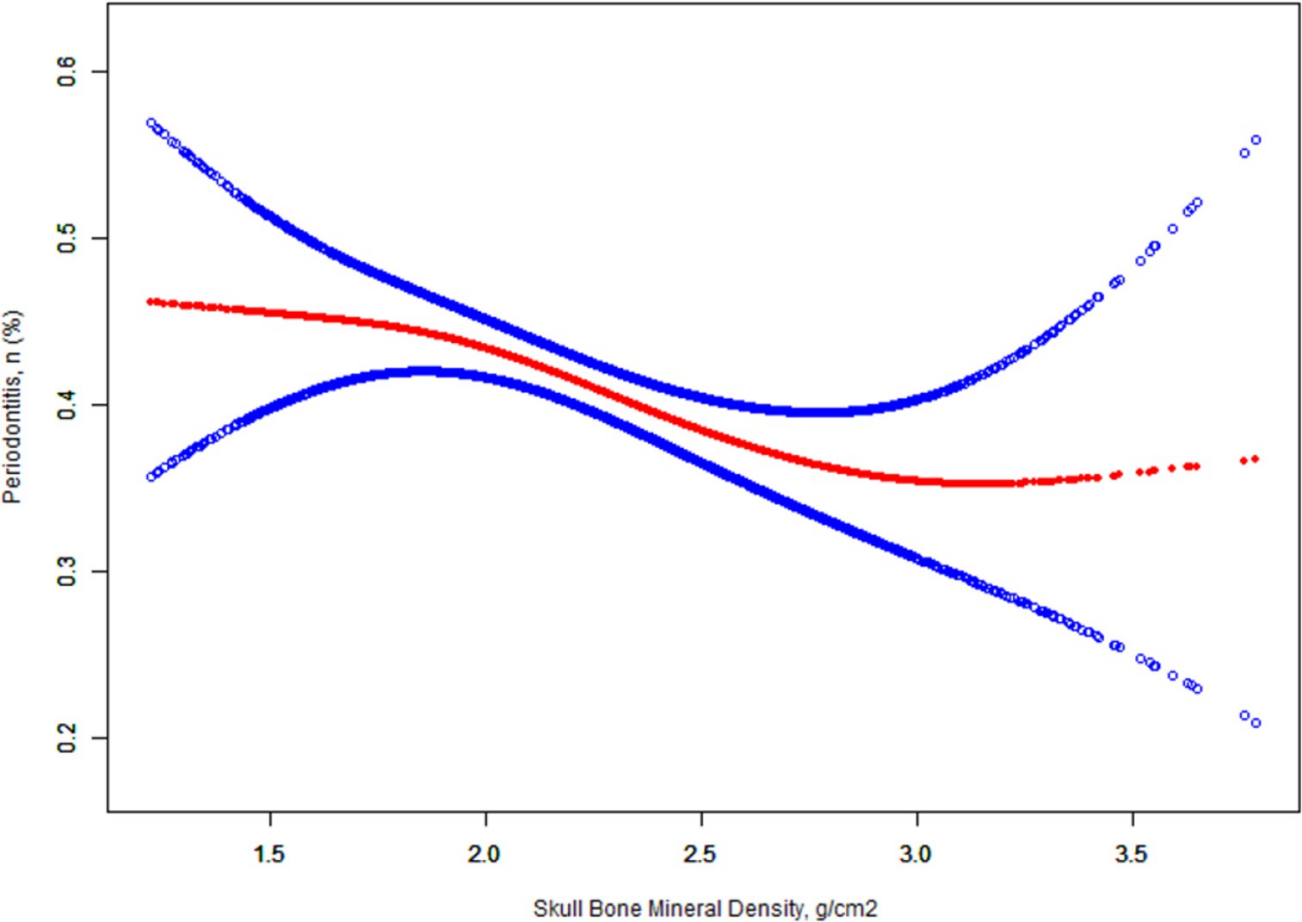
Dose-response relationship between skull BMD and periodontitis*. Solid lines represent smooth curve fitting between variables. The dotted line represents 95 of the fitted confidence interval. *Adjusted for sex; age(smooth); race; education level; PIR; BMI; handgrip strength; smoke consumption; alcohol consumption; hypertension; hypertriglyceridemia; diabetes; general health condition; depressive symptoms; hours of sleep; frequency of interdental hygiene (last 7 days).

The multivariable logistic regression analysis between skull BMD and periodontitis is shown in Table 2. When skull BMD was analyzed as a continuous variable, there was a statistical correlation between skull BMD and periodontitis in the crude model (OR 0.65, 95% CI 0.54–0.77, <0.0001). After adjusting the sociodemographic data, the OR was relatively increased (OR 0.76, 95% CI 0.62–0.93, P = 0.0078). After adjusting all covariates, the OR was relatively decreased compared with model 1 (OR 0.73, 95% CI 0.59–0.90, P = 0.0032). When the skull BMD was assessed as a quartile, in the fully adjusted model, the periodontitis risk of quartile Q2-Q4 was significantly reduced compared with the lowest skull BMD quartile Q1 (Q2: OR 0.95, 95% CI 0.78–1.16, P = 0.6269; Q3: OR 0.78, 95% CI 0.64–0.97, P = 0.0228; Q4: OR 0.70,95% CI 0.56–0.87, P = 0.0014), from the lowest quartile group Q1 to the highest group Q4, the overall trend of all models was consistent (OR 0.88, 95% CI 0.82–0.95, P = 0.0004). In addition, the relationship between skull BMD and periodontitis risk is shown in Fig 1. In general, the risk of periodontitis decreases with the increase of skull BMD. However, when the skull BMD exceeds 2.89 g/cm^2^, the risk of periodontitis will no longer be reduced. Nevertheless, the relationship between skull BMD and periodontitis is still linear, because the threshold effect analysis of skull BMD and periodontitis with piece-wise logistic regression method found that the log likelihood ratio test was 0.152 (P>0.05) (Table 3).

**Table 2:** Association between skull BMD with periodontitis in different models.

Skull BMD, g/cm^2^	OR (95%CI), P-value		
	Crude model	Model Ⅰ	Model Ⅱ
Per 1g/cm2 increase	0.65 (0.54, 0.77) <0.0001	0.76 (0.62, 0.93) 0.0078	0.73 (0.59, 0.90) 0.0032
Skull BMD, (quartile)			
Q1(1.22–1.98)	Ref	Ref	Ref
Q2(1.98–2.21)	0.86 (0.71, 1.03) 0.0939	0.94 (0.77, 1.15) 0.5658	0.95 (0.78, 1.16) 0.6269
Q3(2.21–2.47)	0.70 (0.58, 0.84) 0.0001	0.81 (0.66, 0.99) 0.0414	0.78 (0.64, 0.97) 0.0228
Q4(2.47–3.79)	0.64 (0.53, 0.77) <0.0001	0.72 (0.58, 0.89) 0.0028	0.70 (0.56, 0.87) 0.0014
P for trend	0.86 (0.81, 0.91) <0.0001	0.89 (0.83, 0.96) 0.0012	0.88 (0.82, 0.95) 0.0004

Abbreviations: BMD, bone mineral density. CI, confidence interval. OR, odds ratio.

Crude model adjust for: None.

Model Ⅰ adjust for: sex; age(smooth); race; education level; PIR.

Model Ⅱ adjust for: sex; age(smooth); race; education level; PIR; BMI; handgrip strength; smoke consumption; alcohol consumption; hypertension; hypertriglyceridemia; diabetes; general health condition; depressive symptoms; hours of sleep; frequency of interdental hygiene (last 7 days).

^#^Periodontitis contained mild, moderate, and severe periodontitis.

**Table 3:** Threshold effect analysis of the skull bone mineral density and periodontitis using piece-wise logistic regression.

Skull BMD, g/cm^2^	Periodontitis (OR, 95%CI, P)
Fitting by weighted logistic regression model	0.73 (0.59, 0.90) 0.0036
Fitting by weighted two-piecewise linear logistic mode	
Inflection point	2.89
< 2.89	0.68 (0.54, 0.86) 0.0012
≥ 2.89	1.77 (0.53, 5.93) 0.3541
Log likelihood ratio test	0.149

^#^Periodontitis contained mild, moderate, and severe periodontitis.

## Subgroup analyses

**Fig 2:**
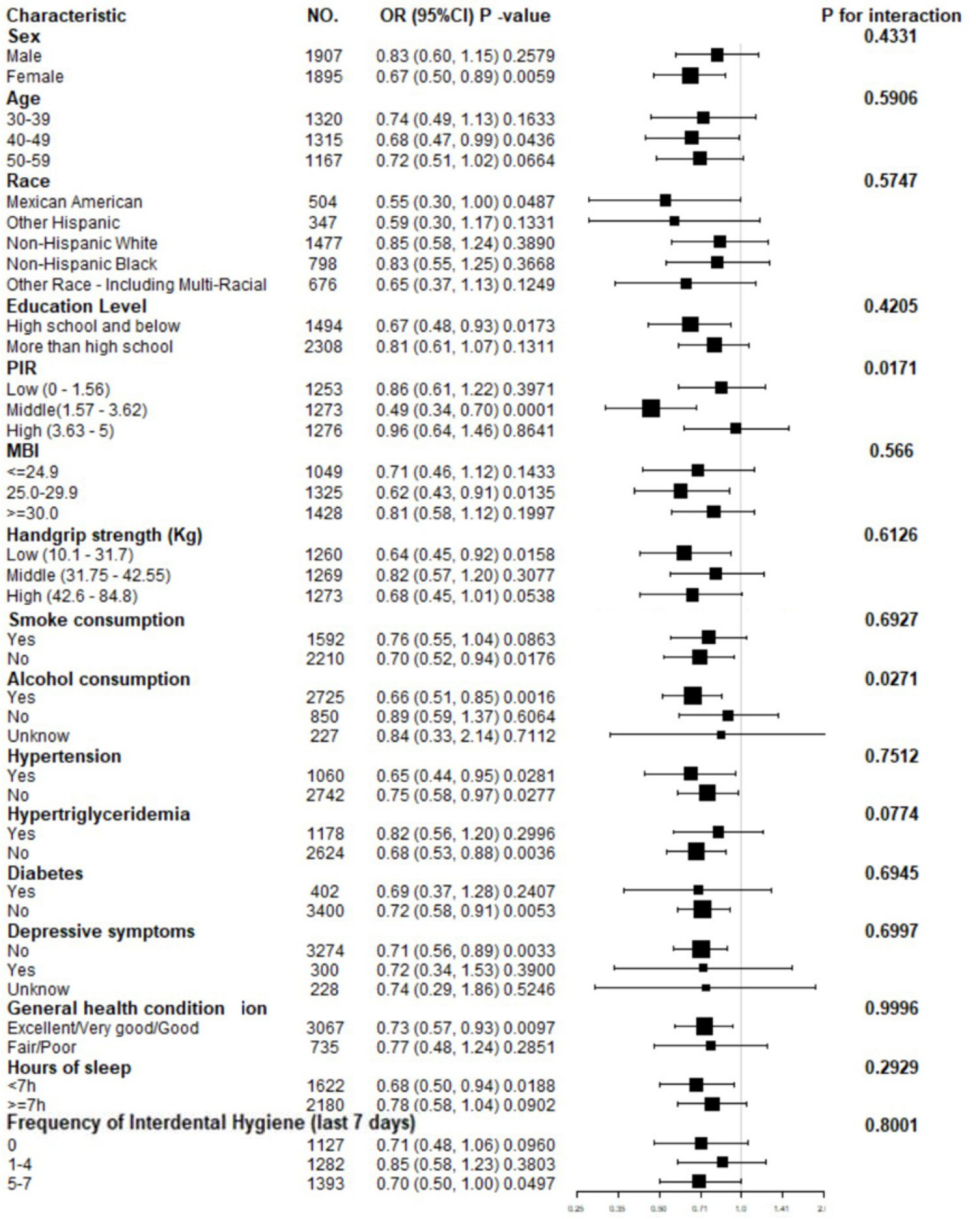
Forest map-Subgroup analyses of the effect of skull BMD on periodontitis*. Abbreviations: PIR, poverty income ratio. BMI, body mass index. CI, confidence interval. OR, odds ratio. *Adjusted for sex, age, race, education level, PIR, BMI, handgrip strength, smoke consumption, alcohol consumption, hypertension, hypertriglyceridemia, diabetes, depressive symptoms, general health condition, hours of sleep, frequency of interdental hygiene (last 7 days) except the subgroup variable.

In the subgroup analysis, we further explored covariates that may affect the relationship between skull BMD and periodontitis, as shown in Fig 2. Subgroup analysis showed that skull BMD and periodontitis remained negative correlation. The influence of skull BMD on periodontitis is more significant in the middle PIR (1.57–3.62) population (Low: OR 0.86, 95% CI 0.61–1.22, P = 0.3971; Middle: OR 0.49, 95% CI 0.34–0.70, P = 0.0001; High: OR 0.96, 95% CI 0.64–1.46, P = 0.8641; P for interaction = 0.0171). Compared with those who had less than 12 alcohol drinks in the past year (OR 0.89, 95% CI0.59–1.37, P = 0.6064), those who had more than 12 alcohol drinks had a lower risk of periodontitis (OR 0.66, 95% CI 0.51–0.85, P = 0.0016, P for interaction = 0.0271). Other covariates had no statistically significant interaction on the association between skull bone density and periodontitis.

## Discussion

In this large cross-sectional study of NHANES 2011–2014, we included a total of 3802 participants, aged 30–59 years. After adjusting other covariates, skull BMD was negatively correlated with periodontitis independently. In addition, we found a linear relationship between skull BMD and periodontitis. Higher skull BMD (Q4: 2.47–3.79g/cm^2^) was associated with a lower risk of periodontitis compared to lower skull BMD (Q1: 1.22–1.98g/cm^2^). In addition, it is proved that the negative correlation between skull BMD and periodontitis is more obvious in the moderate PIR (1.57–3.62) and who had more than 12 alcohol drinks in the past year. As one of the socioeconomic position indicators, PIR has been proved to affect periodontal health [27]. Interestingly, we found that drinking alcohol plays an important role in skull bone mineral density and periodontitis. It is well known that drinking alcohol is an important risk factor for periodontitis [28–30]. On the other hand, low to moderate alcohol intake is associated with slowing down age-related bone loss by reducing the overall rate of bone remodeling [31].

Preclinical studies in ovariectomized animals may explain the link between the low skull BMD and periodontitis. Chen et al. [32] performed ovariectomized mice model, which reliably produced osteopenic or osteoporotic phenotype in long bones, successfully constructed the jaw osteoporotic phenotype, 8 weeks later, it was found that the alveolar bone of osteoporotic mice was significantly thinner, along with periodontal ligament(PDL) width and cell density in the PDL were significantly reduced 18% and 25%, respectively, and a reduced number of osteoprogenitor cells, which persisted even after injury, impacted the rate of alveolar bone regeneration. Likewise, Arioka et al. [33] found the same result. Osteoporotic changes in the periodontium seems to create a vicious circle, leading to more severe periodontitis.

Postmenopausal women with osteoporosis are more susceptible to periodontal disease [6–9], which the consequence of estrogen and BMD could be inferred [8]. Hunziker et al. [34] suggest that declining estrogen secretion can result in a decrease in mandibular BMD in periodontitis patients. The average age of menopause varies by race and lifestyle, but it is around 50 years in most countries [35]. In our study, however, it was not found that the prevalence of periodontitis was higher in females over 50 years old than it was in males. In line with the literature [18], we observed higher skull BMD in woman than in either of the groups of man. The study of Obrant et al. [18] found that women aged 18 to 87 years had significantly higher skull BMD than men of the same age. Wells et al. [36] indicated that women and men have the same bone mineral content in their trunks and limbs before they turn 16, however, from about this age, bone mineral content in all body regions was significantly different between males and females, especially in men, therefore, adult men’s skull bone mass is a small portion of their total bone mass. Paschall et al. [37] investigated the suitability of measuring the skull BMD for estimating age in older adults using DXA and found that a steady increase in the skull BMD in women occurs until they reach 55 years old, at which point an abrupt decline has occurred, in contrast, BMD values in males decrease slightly around 75 years old, but remain steady throughout life. The reason for this difference is the skull BMD did not change synchronously with the rest of the skeleton. Meanwhile, because skull BMD makes up most of the whole body’s BMD, when it is excluded from the whole-body BMD, fracture risk can be better predicted [38, 39].

Furthermore, the mandibular inferior cortex such as mental index (MI) is a part of the panoramic radiograph that can be used to detect osteoporosis in asymptomatic individuals [40, 41]. The results of studies indicate that postmenopausal female dental patients who have MI less than 3 mm on panoramic radiographs, osteoporosis or lower skeletal BMD may be a risk [42, 43]. Notably, instead of being diagnostic criteria, these clinical indexes tend to warn about osteoporosis. Additionally, it was not found that these index values correlated with either the maxillary or mandibular BMD [15], nor with periodontitis [44].

Multiple mediators may have systemic effects on bone formation and bone resorption, the proinflammatory cytokine TNF- α、 IL-1 and IL-6 have been shown to be increased in patients with periodontitis [4]. In which TNF- α and IL-1 have been shown to affect the number and activity of osteoclasts and directly affect osteoblasts to cause cytoplasmic contraction and secretion of collagenase, tissue plasminogen activator [45]. IL—6 increases osteoclast differentiation and leads to osteoblast contraction, making the bone surface more susceptible to resorption [46]. Increased resorption by osteoclasts and decreased proliferation by osteoblasts directly contribute to the decrease in bone mineral density [45]. Thus, an imbalance of pro-inflammatory immune responses may underscore the bidirectional link between periodontitis and systemic and skull low bone mineral density.

Although the 2012 AAP/CDC periodontitis case definition was used in the past as a global standard in epidemiological studies of periodontal disease [26, 47], in 2018 the European Federation of Periodontology/American Academy of Periodontology (EFP/AAP) issued a new classification of periodontal disease and called for the ability to replace the 2012 CDC/AAP periodontitis case definition [48, 49]. The 2018 EFP / AAP periodontal disease classification stages and grades periodontitis based on severity, complexity and disease progression rate. In terms of staging, 1–2, 3–4, and ≥ 5 mm of the site of maximum loss are considered to be incipient (stage 1), moderate (stage 2), and severe (stages 3 and 4), respectively [48]. Although CAL is accepted as the gold standard for the severity and progression of periodontitis, it may be difficult to distinguish initial periodontitis from gingivitis when used alone [47]. In addition, there are non-periodontal inflammatory factors that can cause CAL such as: traumatic gingival recession; and caries extending to the neck of the tooth, etc [48]. However, the cause of CAL is not specifically described in the NHANES periodontitis database. Therefore, analysis of NHANES data using the 2018 EFP/AAP classification of periodontal diseases may increase the prevalence of periodontitis [50, 51]. CAL and periodontal PD were used in the 2012 AAP/CDC periodontitis case definition, minimizing the potential erroneous effect of periodontal recession on the accuracy of probing depth measurements [47]. Nevertheless, the 2018 EFP/AAP classification of periodontal diseases provides more detailed staging and grading of periodontitis, and future epidemiological investigations of periodontitis should document more detailed periodontal status.

According to our study, this is the first study using NHANES database to evaluate the relationship between skull BMD and periodontitis. In addition, our study included craniofacial bone, and the most ideal method (dual energy X-ray absorption method) was used to detect BMD. However, despite these advantages, there are also some limitations. First of all, this is a cross-sectional study, and causality cannot be confirmed. Secondly, NHANES 2011–2012 does not include all confounding factors. For example, people with high BMD are not distinguished, because the increase of BMD may also be secondary to a series of potential diseases affecting bones. Finally, due to the limited research on skull BMD and periodontitis, this study cannot be compared with other studies, resulting in a limited scope of discussion. Therefore, this study can not reflect the most true relationship between skull BMD and periodontitis, and a longitudinal study should be conducted in the future considering more relevant variables of skull BMD and periodontitis.

## Conclusion

This result suggested that periodontal disease can be related to low skull BMD, for those people, oral hygiene and health care should be more closely monitored. Validation of our findings will require further research.

## References

[1] Wu L, Zhang SQ, Zhao L, Ren ZH, Hu CY. Global, regional, and national burden of periodontitis from 1990 to 2019: Results from the Global Burden of Disease study 2019. Journal of periodontology. 2022. Epub 2022/03/20. doi: 10.1002/JPER.21-0469 . 35305266

[2] Pihlstrom BL, Michalowicz BS, Johnson NW. Periodontal diseases. Lancet (London, England). 2005;366(9499):1809–20. Epub 2005/11/22. doi: 10.1016/S0140-6736(05)67728-8 . 16298220

[3] Passos JS, Vianna MI, Gomes-Filho IS, Cruz SS, Barreto ML, Adan L, et al. Osteoporosis/osteopenia as an independent factor associated with periodontitis in postmenopausal women: a case-control study. Osteoporosis international: a journal established as result of cooperation between the European Foundation for Osteoporosis and the National Osteoporosis Foundation of the USA. 2013;24(4):1275–83. Epub 2012/09/25. doi: 10.1007/s00198-012-2130-7 . 23001114

[4] Cardoso EM, Reis C, Manzanares-Céspedes MC. Chronic periodontitis, inflammatory cytokines, and interrelationship with other chronic diseases. Postgraduate medicine. 2018;130(1):98–104. Epub 2017/10/27. doi: 10.1080/00325481.2018.1396876 . 29065749

[5] Oztürk Tonguç M, Büyükkaplan US, Fentoglu O, Gümüs BA, Çerçi SS, Kirzioglu FY. Comparison of bone mineral density in the jaws of patients with and without chronic periodontitis. Dento maxillo facial radiology. 2012;41(6):509–14. Epub 2012/01/14. doi: 10.1259/dmfr/21900076 ; PubMed Central PMCID: PMC3520387. 22241867 PMC3520387

[6] Kim EJ, Lim CH, Eun M, Yu SA, Kwon SM, Lee JE, et al. Association between Dental Caries and Handgrip Strength: In a Population-Based Study in Korea (KNHANES 2016–2018). International journal of environmental research and public health. 2022;19(16). Epub 2022/08/27. doi: 10.3390/ijerph19169874 ; PubMed Central PMCID: PMC9408064. 36011504 PMC9408064

[7] Hong SJ, Yang BE, Yoo DM, Kim SJ, Choi HG, Byun SH. Analysis of the relationship between periodontitis and osteoporosis/fractures: a cross-sectional study. BMC oral health. 2021;21(1):125. Epub 2021/03/19. doi: 10.1186/s12903-021-01496-1 ; PubMed Central PMCID: PMC7968237. 33731091 PMC7968237

[8] Kim CS, Kim EK, Lee KS, Lee HK, Choi YH, Hwang TY, et al. Relationship between bone mineral density, its associated physiological factors, and tooth loss in postmenopausal Korean women. BMC women’s health. 2015;15:65. Epub 2015/08/27. doi: 10.1186/s12905-015-0218-x ; PubMed Central PMCID: PMC4549858. 26306548 PMC4549858

[9] Kim JW, Kong KA, Kim HY, Lee HS, Kim SJ, Lee SH, et al. The association between bone mineral density and periodontitis in Korean adults (KNHANES 2008–2010). Oral Dis. 2014;20(6):609–15. Epub 2013/10/15. doi: 10.1111/odi.12179 . 24118189

[10] Yu B, Wang CY. Osteoporosis and periodontal diseases—An update on their association and mechanistic links. Periodontology 2000. 2022;89(1):99–113. Epub 2022/03/05. doi: 10.1111/prd.12422 ; PubMed Central PMCID: PMC9067601. 35244945 PMC9067601

[11] Sun Y, An MM, Wang J, Yang F, He L, Dudzek CA, et al. Assessment of the Bone Mineral Content in the Mandible by Dual-Energy X-Ray Absorptiometry to Evaluate Short-Term Change. Journal of clinical densitometry: the official journal of the International Society for Clinical Densitometry. 2018;21(4):534–40. Epub 2017/08/07. doi: 10.1016/j.jocd.2017.06.003 . 28781228

[12] Suzuki F, Okamoto S, Miyagi S, Tsujiguchi H, Hara A, Nguyen TTT, et al. Relationship between Decreased Mineral Intake Due to Oral Frailty and Bone Mineral Density: Findings from Shika Study. Nutrients. 2021;13(4). Epub 2021/05/01. doi: 10.3390/nu13041193 ; PubMed Central PMCID: PMC8066385. 33916336 PMC8066385

[13] Barr C, Sharafieh R, Schwarz G, Wu R, Klueh U, Kreutzer D. Noninflammatory Stress-Induced Remodeling of Mandibular Bone: Impact of Age and Pregnancy. Journal of oral and maxillofacial surgery: official journal of the American Association of Oral and Maxillofacial Surgeons. 2021;79(5):1147–55. Epub 2021/01/08. doi: 10.1016/j.joms.2020.12.003 . 33412113

[14] Munhoz L, Morita L, Nagai AY, Moreira J, Arita ES. Mandibular cortical index in the screening of postmenopausal at low mineral density risk: a systematic review. Dento maxillo facial radiology. 2021;50(4):20200514. Epub 2021/02/17. doi: 10.1259/dmfr.20200514 ; PubMed Central PMCID: PMC8078000. 33591840 PMC8078000

[15] Gulsahi A, Paksoy CS, Ozden S, Kucuk NO, Cebeci AR, Genc Y. Assessment of bone mineral density in the jaws and its relationship to radiomorphometric indices. Dento maxillo facial radiology. 2010;39(5):284–9. Epub 2010/07/01. doi: 10.1259/dmfr/20522657 ; PubMed Central PMCID: PMC3520251. 20587652 PMC3520251

[16] Herring S, Ochareon P. Bone–special problems of the craniofacial region. Orthodontics & craniofacial research. 2005;8(3):174–82. doi: 10.1111/j.1601-6343.2005.00328.x 16022719

[17] Turner AS, Maillet JM, Mallinckrodt C, Cordain L. Bone mineral density of the skull in premenopausal women. Calcified tissue international. 1997;61(2):110–3. Epub 1997/08/01. doi: 10.1007/s002239900305 . 9312398

[18] Obrant K. Bone mineral density in the head is higher in women than in men. Bone. 1995;16:S153.

[19] Courteix D, Lespessailles E, Obert P, Benhamou CL. Skull bone mass deficit in prepubertal highly-trained gymnast girls. International journal of sports medicine. 1999;20(5):328–33. Epub 1999/08/19. doi: 10.1055/s-2007-971139 . 10452231

[20] Taylor A, Waxman K, Izfar S, Grotts J, Yim S. Screening for osteoporosis after trauma: a new approach using quantitative computed tomography of the skull. The journal of trauma and acute care surgery. 2014;77(4):635–9. Epub 2014/09/25. doi: 10.1097/TA.0000000000000411 . 25250607

[21] NCHS Ethics Review Board (ERB) Approval*. Available from: https://www.cdc.gov/nchs/nhanes/irba98.htm.

[22] Liang X, Fan J. The utilization of accurate body mass index classification is imperative for grouping based on BMI. Human reproduction (Oxford, England). 2022;37(3):622–3. Epub 2022/01/01. doi: 10.1093/humrep/deab276 . 34970977

[23] Beydoun HA, Hossain S, Beydoun MA, Weiss J, Zonderman AB, Eid SM. Periodontal disease, sleep duration, and white blood cell markers in the 2009 to 2014 National Health and Nutrition Examination Surveys. Journal of periodontology. 2020;91(5):582–95. Epub 2019/09/26. doi: 10.1002/JPER.19-0055 ; PubMed Central PMCID: PMC7735683. 31554016 PMC7735683

[24] Wiener RC. Relationship of Routine Inadequate Sleep Duration and Periodontitis in a Nationally Representative Sample. Sleep disorders. 2016;2016:9158195. Epub 2016/02/24. doi: 10.1155/2016/9158195 ; PubMed Central PMCID: PMC4745352. 26904296 PMC4745352

[25] Badawy R, El-Mowafy O, Tam LE. Fracture toughness of chairside CAD/CAM materials—Alternative loading approach for compact tension test. Dental materials: official publication of the Academy of Dental Materials. 2016;32(7):847–52. Epub 2016/05/03. doi: 10.1016/j.dental.2016.03.003 . 27133875

[26] Eke PI, Page RC, Wei L, Thornton-Evans G, Genco RJ. Update of the case definitions for population-based surveillance of periodontitis. Journal of periodontology. 2012;83(12):1449–54. Epub 2012/03/17. doi: 10.1902/jop.2012.110664 ; PubMed Central PMCID: PMC6005373. 22420873 PMC6005373

[27] Borrell LN, Crawford ND. Socioeconomic position indicators and periodontitis: examining the evidence. Periodontology 2000. 2012;58(1):69–83. Epub 2011/12/03. doi: 10.1111/j.1600-0757.2011.00416.x ; PubMed Central PMCID: PMC3233193. 22133367 PMC3233193

[28] Baumeister SE, Freuer D, Nolde M, Kocher T, Baurecht H, Khazaei Y, et al. Testing the association between tobacco smoking, alcohol consumption, and risk of periodontitis: A Mendelian randomization study. Journal of clinical periodontology. 2021;48(11):1414–20. Epub 2021/09/03. doi: 10.1111/jcpe.13544 . 34472130

[29] Gay IC, Tran DT, Paquette DW. Alcohol intake and periodontitis in adults aged ≥30 years: NHANES 2009–2012. Journal of periodontology. 2018;89(6):625–34. Epub 2018/03/25. doi: 10.1002/jper.17-0276 . 29572839

[30] Han SJ, Yi YJ, Bae KH. The association between periodontitis and dyslipidemia according to smoking and harmful alcohol use in a representative sample of Korean adults. Clinical oral investigations. 2020;24(2):937–44. Epub 2019/07/05. doi: 10.1007/s00784-019-02989-8 . 31270667

[31] Gaddini GW, Turner RT, Grant KA, Iwaniec UT. Alcohol: a simple nutrient with complex actions on bone in the adult skeleton. Alcoholism: clinical and experimental research. 2016;40(4):657–71. doi: 10.1111/acer.13000 26971854 PMC4918769

[32] Chen CH, Wang L, Serdar Tulu U, Arioka M, Moghim MM, Salmon B, et al. An osteopenic/osteoporotic phenotype delays alveolar bone repair. Bone. 2018;112:212–9. Epub 2018/04/29. doi: 10.1016/j.bone.2018.04.019 . 29704698 PMC13561117

[33] Arioka M, Zhang X, Li Z, Tulu US, Liu Y, Wang L, et al. Osteoporotic Changes in the Periodontium Impair Alveolar Bone Healing. Journal of dental research. 2019;98(4):450–8. Epub 2019/01/11. doi: 10.1177/0022034518818456 ; PubMed Central PMCID: PMC6429667. 30626268 PMC6429667

[34] Hunziker J, Wronski TJ, Miller SC. Mandibular bone formation rates in aged ovariectomized rats treated with anti-resorptive agents alone and in combination with intermittent parathyroid hormone. Journal of dental research. 2000;79(6):1431–8. Epub 2000/07/13. doi: 10.1177/00220345000790061301 . 10890724

[35] Shi J, Zhang B, Choi JY, Gao YT, Li H, Lu W, et al. Age at menarche and age at natural menopause in East Asian women: a genome-wide association study. Age (Dordrecht, Netherlands). 2016;38(5–6):513–23. Epub 2016/09/16. doi: 10.1007/s11357-016-9939-5 ; PubMed Central PMCID: PMC5266214. 27629107 PMC5266214

[36] Wells JC. Sexual dimorphism of body composition. Best practice & research Clinical endocrinology & metabolism. 2007;21(3):415–30. Epub 2007/09/19. doi: 10.1016/j.beem.2007.04.007 . 17875489

[37] Paschall A, Ross AH. Biological sex variation in bone mineral density in the cranium and femur. Science & justice: journal of the Forensic Science Society. 2018;58(4):287–91. Epub 2018/06/14. doi: 10.1016/j.scijus.2018.01.002 . 29895462

[38] Taylor A, Konrad PT, Norman ME, Harcke HT. Total body bone mineral density in young children: influence of head bone mineral density. Journal of Bone and Mineral Research. 1997;12(4):652–5. doi: 10.1359/jbmr.1997.12.4.652 9101377

[39] Ringertz J. Effect of bone density of the head on total body DEXA measurements in 100 healthy Swedish women. Acta radiologica (Stockholm, Sweden: 1987). 1996;37(1):101–6. Epub 1996/01/01. doi: 10.1177/02841851960371P120 . 8611312

[40] Munhoz L, Takahashi DY, Nishimura DA, Ramos E, Tenorio JDR, Arita ES. Do Patients with Osteoporosis Have Higher Risk to Present Reduced Alveolar Ridge Height? An Imaging Analysis. Indian journal of dental research: official publication of Indian Society for Dental Research. 2019;30(5):747–50. Epub 2019/12/20. doi: 10.4103/ijdr.IJDR_497_18 . 31854367

[41] Ren J, Fan H, Yang J, Ling H. Detection of Trabecular Landmarks for Osteoporosis Prescreening in Dental Panoramic Radiographs. Annual International Conference of the IEEE Engineering in Medicine and Biology Society IEEE Engineering in Medicine and Biology Society Annual International Conference. 2020;2020:2194–7. Epub 2020/10/07. doi: 10.1109/EMBC44109.2020.9175281 . 33018442

[42] Taguchi A, Tanaka R, Kakimoto N, Morimoto Y, Arai Y, Hayashi T, et al. Clinical guidelines for the application of panoramic radiographs in screening for osteoporosis. Oral Radiol. 2021;37(2):189–208. Epub 2021/02/24. doi: 10.1007/s11282-021-00518-6 . 33620644

[43] Jonasson G, Hassani-Nejad A, Hakeberg M. Mandibular cortical bone structure as risk indicator in fractured and non-fractured 80-year-old men and women. BMC oral health. 2021;21(1):468. Epub 2021/09/26. doi: 10.1186/s12903-021-01829-0 ; PubMed Central PMCID: PMC8461912. 34560860 PMC8461912

[44] Mudda JA, Bajaj M, Patil VA. A Radiographic comparison of mandibular bone quality in pre- and post-menopausal women in Indian population. Journal of Indian Society of Periodontology. 2010;14(2):121–5. Epub 2010/04/01. doi: 10.4103/0972-124X.70833 ; PubMed Central PMCID: PMC3110466. 21691550 PMC3110466

[45] Hienz SA, Paliwal S, Ivanovski S. Mechanisms of Bone Resorption in Periodontitis. Journal of immunology research. 2015;2015:615486. Epub 2015/06/13. doi: 10.1155/2015/615486 ; PubMed Central PMCID: PMC4433701. 26065002 PMC4433701

[46] Apolinário Vieira GH, Aparecida Rivas AC, Figueiredo Costa K, Ferreira Oliveira LF, Tanaka Suzuki K, Reis Messora M, et al. Specific inhibition of IL-6 receptor attenuates inflammatory bone loss in experimental periodontitis. Journal of periodontology. 2021;92(10):1460–9. Epub 2021/01/26. doi: 10.1002/JPER.20-0455 . 33492708

[47] Eke PI, Borgnakke WS, Genco RJ. Recent epidemiologic trends in periodontitis in the USA. Periodontology 2000. 2020;82(1):257–67. Epub 2019/12/19. doi: 10.1111/prd.12323 . 31850640

[48] Tonetti MS, Greenwell H, Kornman KS. Staging and grading of periodontitis: Framework and proposal of a new classification and case definition. Journal of periodontology. 2018;89 Suppl 1:S159–s72. Epub 2018/06/22. doi: 10.1002/jper.18-0006 . 29926952

[49] Botelho J, Machado V, Proença L, Mendes JJ. The 2018 periodontitis case definition improves accuracy performance of full-mouth partial diagnostic protocols. Scientific reports. 2020;10(1):7093. Epub 2020/04/29. doi: 10.1038/s41598-020-63700-6 ; PubMed Central PMCID: PMC7184582. 32341429 PMC7184582

[50] Germen M, Baser U, Lacin CC, Fıratlı E, İşsever H, Yalcin F. Periodontitis Prevalence, Severity, and Risk Factors: A Comparison of the AAP/CDC Case Definition and the EFP/AAP Classification. International journal of environmental research and public health. 2021;18(7). Epub 2021/04/04. doi: 10.3390/ijerph18073459 ; PubMed Central PMCID: PMC8037399. 33810461 PMC8037399

[51] Costea CA, Christodorescu R, Soancă A, Roman A, Micu IC, Stratul Ș I, et al. Periodontitis in Ischemic Stroke Patients: Case Definition Challenges of the New Classification Scheme (2018). Journal of clinical medicine. 2022;11(3). Epub 2022/02/16. doi: 10.3390/jcm11030520 ; PubMed Central PMCID: PMC8836590. 35159973 PMC8836590

